# First Report of* Eurycoma longifolia* Jack Root Extract Causing Relaxation of Aortic Rings in Rats

**DOI:** 10.1155/2016/1361508

**Published:** 2016-10-09

**Authors:** Bae Huey Tee, See Ziau Hoe, Swee Hung Cheah, Sau Kuen Lam

**Affiliations:** Department of Physiology, Faculty of Medicine, University of Malaya, 50603 Kuala Lumpur, Malaysia

## Abstract

Although* Eurycoma longifolia* has been studied for erectile function, the blood pressure- (BP-) lowering effect has yet to be verified. Hence, this study aims at investigating the BP-lowering properties of the plant with a view to develop an antihypertensive agent that could also preserve erectile function. Ethanolic root extract was partitioned by hexane, dichloromethane (DCM), ethyl acetate, butanol, and water. The DCM fraction, found to be potent in relaxing phenylephrine- (PE-) precontracted rat aortic rings, was further purified by column chromatography. Subfraction DCM-II, being the most active in relaxing aortae, was studied for effects on the renin-angiotensin and kallikrein-kinin systems in aortic rings. The effect of DCM-II on angiotensin-converting enzyme (ACE) activity was also evaluated* in vitro*. Results showed that DCM-II reduced (*p* < 0.05) the contractions evoked by angiotensin I and angiotensin II (Ang II). In PE-precontracted rings treated with DCM-II, the Ang II-induced contraction was attenuated (*p* < 0.05) while bradykinin- (BK-) induced relaxation enhanced (*p* < 0.001).* In vitro*, DCM-II inhibited (*p* < 0.001) the activity of ACE. These data demonstrate that the vasodilatory effect of DCM-II appears to be mediated* via* inhibition of Ang II type 1 receptor and ACE as well as enhancement of Ang II type 2 receptor activation and BK activity.

## 1. Introduction

*Eurycoma longifolia* (*E. longifolia*) Jack, a shrub tree that originates from South-East Asia [1], has been conferred a myriad of medicinal properties supposedly efficacious or otherwise. Thus, in Vietnam, it is known as* cay ba binh *which means the tree that cures hundreds of diseases [2], while in Malaysia, the root of this plant is deemed to be Malaysian ginseng [3], with a reputation of being able to enhance male sexual functions [2]. Traditionally, decoction or tincture of the root [4] is also used to enhance the immune system [2] and treat ulcer [1], malaria, and hypertension [2]. Numerous scientific investigations have been conducted to verify the purported therapeutic claims of* E. longifolia *with findings showing that it indeed does possess antiulcer [5] and antimalarial [6–8] activities as well as being able to improve sexual functions [9–14]. However, effects of this plant on blood pressure (BP) have yet to be explored.

Normal BP is partly maintained by the renin-angiotensin system (RAS), a key component of the homeostatic mechanisms of the body [15]. In the RAS, angiotensin-converting enzyme (ACE) cleaves a dipeptide from the C-terminal of angiotensin I (Ang I) to produce angiotensin II (Ang II) [16] that causes a prolonged increase in BP due to vasoconstriction upon stimulation of the angiotensin II type 1 receptor (AT_1_R) [17]. In addition, ACE which is also known as kininase II breaks down bradykinin (BK), a peptide produced in the kallikrein-kinin system that reduces BP by promoting vasodilation, natriuresis, and diuresis [18], into an inactive peptide by sequential cleaving of two dipeptides from the C-terminal [19]. Thus, AT_1_R blockers (ARBs) as well as ACE inhibitors (ACEi) that reduce the formation of Ang II and decrease hydrolysis of BK are the cornerstones in the treatment of hypertension [20], as enhancing vascular smooth muscle (VSM) relaxation may invariably bring about vasodilation and consequently lower BP.

Hypertension, the presence of chronic elevation of BP above 140/90 mmHg [21], is estimated to affect 1.56 billion adults by 2025, an increase from 972 million in 2000 [22]. Despite being cognizant of increased risks of cardiovascular and kidney diseases [23] with hypertension, total compliance with adherence to conventional treatment among hypertensive individuals still poses a challenge for healthcare providers. This could be due to the various side-effects experienced by the patients ranging from mild to serious, and one particularly perplexing side-effect is erectile dysfunction (ED) [24, 25] which greatly affects their quality of life [26].

In view of the reported androgenic and/or erectogenic properties [9, 11, 13] as well as the anecdotal hypotensive effects of* E. longifolia*, it will be of particular interest to investigate the hypotensive properties of this plant with a perspective of developing antihypertensive agents that not only do they not give rise to ED but also improve erectile function. Taking into consideration that vascular tone and RAS are critical in the regulation of BP, the present study is conducted to investigate the effect of* E. longifolia *on the contractile activity of VSM as well as the possibilities of* E. longifolia *of modulating local vascular RAS to bring about relaxation in the VSM.

## 2. Materials and Methods

### 2.1. Materials

#### 2.1.1. Plant Materials

Roots of* E. longifolia* were collected from Peninsular Malaysia, with a specimen (number: KLU 47214) being authenticated and deposited in the Herbarium at the* Rimba Ilmu*, University of Malaya.

#### 2.1.2. Animals

Adult male Sprague-Dawley (SD) rats aged 2-3 months and weighing 200–210 g were obtained from the Animal Experimental Unit, University of Malaya. All the animals were maintained and used according to the international standards found in the* Guide for the Care and Use of Laboratory Animals *[27]. They were kept under standard conditions with clean water and standard rat chow (Altromin, Eastern-Westphalia, Germany) provided* ad libitum*. All experimental procedures that involve animals were approved by the University of Malaya Animal Care and Ethics Committee (Ethics Reference number: 2014-01-07/PHYSIO/R/TBH).

#### 2.1.3. Chemicals and Drugs

All chemicals used were of analytical grade and purchased from Merck KGaA (Darmstadt, Germany) except for 95% aqueous ethanol which was purchased from R & M Marketing (Essex, UK). Phenylephrine (PE) hydrochloride, acetylcholine (ACh) chloride, Ang I, Ang II, BK, ACE, hippuryl-L-histidyl-L-leucine (HHL), and Tween® 80 were from Sigma-Aldrich Co. (St. Louis, MO, USA). Antagonists of angiotensin II type 2 receptor (AT_2_R), PD 123319, and Mas receptor, A-779, were from Bachem (Bubendorf, Switzerland) and Tocris Bioscience (Bristol, UK), respectively.

### 2.2. Methods

#### 2.2.1. Extraction and Purification of Plant Materials

Fresh roots were cleaned and dried before being pulverised to powder. Then, the powder was macerated with 95% aqueous ethanol. The crude ethanolic extract was obtained after the ethanol was evaporated to dryness in a rotary evaporator.

The crude ethanolic extract was reconstituted in distilled water so as to be partitioned with organic solvent, hexane (HX), in a separating funnel. The upper HX layer was collected and then evaporated to dryness* in vacuo* to obtain the HX fraction. The bottom aqueous layer was further partitioned with another organic solvent, dichloromethane (DCM), to obtain the DCM fraction. Similarly, the remaining aqueous layer was partitioned sequentially with ethyl acetate (EA) and then water-saturated butanol to obtain the EA and water-saturated butanolic (BU) fractions, respectively. Finally, the aqueous layer was lyophilised to obtain the final aqueous (FA) fraction. In order to ascertain the fraction that has effect on VSM, the relaxant activity of each fraction was studied on the aortic ring (refer to Section 2.2.2(2)) to obtain the most potent fraction. The DCM fraction (refer to Section 3.2.1) which was found to fulfil the criteria was then subjected to further purification.

The DCM fraction was loaded onto a column packed with Merck silica gel 60 (15–40 *μ*m) and then eluted stepwise with 6 mixtures of organic solvents in increasing ratios (by 10%) of polar solvents, starting with a mixture of EA and HX (75 : 25) to obtain DCM-I, the first DCM subfraction. This was then followed by another 2 cycles of elution using EA and HX mixtures of ratios 85 : 15 and 95 : 5 to obtain DCM-II and DCM-III, respectively. Subsequently, the column was eluted with mixtures of EA and methanol with ratios of 95 : 5, 85 : 15, and 75 : 25 in order to obtain DCM-IV, DCM-V, and DCM-VI, respectively. Once again, each DCM subfraction was tested for relaxant activity on aortic rings (refer to Section 2.2.2(2)) to obtain the most potent subfraction. The DCM-II (refer to Section 3.2.2) which was found to be the most effective subfraction was then used for studies on the mechanisms of action in aortic rings.

#### 2.2.2. *Ex Vivo *Studies on Isolated Aortic Ring

*(1) Preparation and Validation of Aortic Ring*. Preparation and validation of aortic rings were carried out according to the method of Hoe et al. [28]. Rats were sacrificed by cervical dislocation. From each rat, the descending thoracic aorta was dissected out and immediately placed in chilled, 95% oxygenated Krebs-bicarbonate solution containing 118.1 mM NaCl, 4.7 mM KCl, 1.0 mM KH_2_PO_4_, 1.0 mM MgSO_4_, 25.0 mM NaHCO_3_, 2.5 mM CaCl_2_, and 11.1 mM glucose. The aorta was carefully cleaned of adhering fat and connective tissue and then cut into rings of 3 mm length. To prepare the denuded aortic ring, a pair of fine forceps was inserted into the lumen and the aorta was gently rotated around the forceps. After that, the aortic ring was mounted in a tissue chamber by inserting two parallel L-shaped stainless steel holders, with one end being the tissue holder and the other end being hooked to a force transducer connected to a physiological data acquisition system (PowerLab®; ADInstruments Pty Ltd., Bella Vista, Australia). The bath solution in the tissue chamber was maintained at 37°C and aerated with 95% oxygen and 5% carbon dioxide. Each aortic ring was allowed to equilibrate for 60 minutes at an optimum resting tension (1 g) with changing of bath fluid every 15 minutes before commencement of all experiments. In addition, each aortic ring was stimulated with KCl (6 × 10^−2 ^M) at least three times before each experiment to ensure that the contractile response was reproducible. The intactness of endothelium was determined by adding ACh (1 × 10^−6 ^M) to PE-precontracted (1 × 10^−6 ^M) aortic ring. Aortic rings that were able to relax ≥70% of the maximal contraction indicated that the endothelia were intact while an absence of relaxation denoted that the endothelia were satisfactorily denuded. At the end of each dose-response experiment, the preparation was tested with PE so as to assure that the tissue was still viable.

*(2) Effects of Partitioned (HX, DCM, EA, BU, and FA) Fractions and DCM (DCM-I to DCM-VI) Subfractions on PE-Precontracted Aortic Ring*. Aortic rings were precontracted with the addition of PE (1 × 10^−6 ^M) in the tissue chamber. After a stable contractile response had been achieved, increasing doses of each of the partitioned (HX, DCM, EA, BU, and FA) fractions (0.1–3 mg/mL) or DCM (DCM-I to DCM-VI) subfractions (0.02–0.08 mg/mL) were added cumulatively to the tissue chamber.

#### 2.2.3. Mechanisms of Action of DCM-II on Aortic Ring

*(1) Effects of DCM-II on Ang I- or Ang II-Induced Contractions*. Aortic rings were pretreated with DCM-II (0.08 mg/mL) or 0.15% Tween 80 in distilled water (vehicle). After 15 minutes of incubation period, increasing doses of Ang I or Ang II (3.16 × 10^−9^–3.16 × 10^−6 ^M) were added cumulatively.

*(2) Effect of DCM-II on Ang II-Induced Contractions in PE-Precontracted Aortic Ring*. This study was conducted according to the method of Arun et al. [29]. Aortic rings were pretreated with DCM-II (0.08 mg/mL) or vehicle. After 15 minutes of treatment, PE (1 × 10^−6 ^M) was added to precontract the aortic rings. Once a stable contractile response had been achieved, increasing doses of Ang II (3.16 × 10^−9^–3.16 × 10^−6 ^M) were added cumulatively.

*(3) Effects of Antagonists of AT*_*2*_ *R (PD 123319) and Mas Receptor (A-779) on Ang II-Induced Relaxation in PE-Precontracted Aortic Rings in the Presence of DCM-II*. Aortic rings were preincubated with DCM-II (0.08 mg/mL) in combination with antagonists of either AT_2_R (PD 123319; 1 × 10^−7 ^M), Mas receptor (A-779; 1 × 10^−6 ^M), or vehicle. After 15 minutes of incubation, PE (1 × 10^−6 ^M) was added to precontract the rings. Once a stable contractile response had been achieved, Ang II (3.16 × 10^−6 ^M) was added to the tissue chamber. 

*(4) Effect of DCM-II on BK-Induced Relaxations in PE-Precontracted Aortic Ring*. Aortic rings were incubated with DCM-II (0.08 mg/mL) or vehicle for 15 minutes, after which PE (1 × 10^−6 ^M) was added to precontract the aortic rings. Increasing doses of BK (3.16 × 10^−7^–3.16 × 10^−5 ^M) were added cumulatively once the PE-induced contraction had stabilised.

#### 2.2.4. *In Vitro* Studies

*(1) Effect of DCM-II on ACE Activity*. The effect of DCM-II on activity of ACE was studied according to the method of Hoe et al. [30]. Briefly, DCM-II (0.01–0.08 mg/mL) or vehicle was added to the incubation buffer containing ACE and the substrate, HHL. In this assay, hippurate produced from the cleavage of HHL by ACE was mixed with cyanuric chloride to form chromogens that were quantified by measuring the absorbance at 382 nm. Blanks were assayed similarly except that the terminating solution, hydrochloric acid, was added before the addition of substrate.

#### 2.2.5. Statistical Analyses

Values are expressed as mean ± SEM. Data were analysed by SPSS® Statistics version 17.0 (SPSS Inc., Chicago, IL, USA) and graphed using GraphPad Prism® version 5 for Windows (GraphPad Software Inc., San Diego, California, USA). The differences between two groups were evaluated by Student's* t*-test while multiple group comparisons were performed by one-way ANOVA Tukey's test. A probability value of less than 0.05 (*p* < 0.05) was considered to be statistically significant.

## 3. Results

### 3.1. Extraction and Purification of Plant Materials

Partition of the ethanolic root extracts of* E. longifolia* produced 5 fractions (HX, DCM, EA, BU, and FA) and the subsequent fractionation of DCM produced 6 subfractions (DCM-I to DCM-VI) for further investigations.

### 3.2. *Ex Vivo *Studies on Isolated Aortic Ring

#### 3.2.1. Effects of Partitioned (HX, DCM, EA, BU, and FA) Fractions on PE-Precontracted Aortic Ring

**Figure 1:**
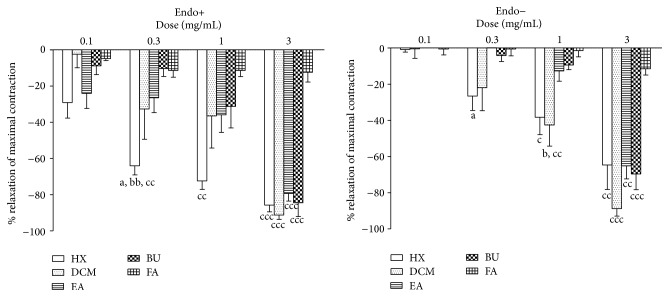
Effects of partitioned fractions on PE-precontracted endothelium-intact (Endo+) and endothelium-denuded (Endo−) aortic rings. Values are mean ± SEM (*n* = 6). ^a^ *p* < 0.05 compared with EA, ^b^ *p* < 0.05, ^bb^ *p* < 0.01 compared with BU, ^c^ *p* < 0.05, ^cc^ *p* < 0.01, and ^ccc^ *p* < 0.001 compared with FA.

**Figure 2:**
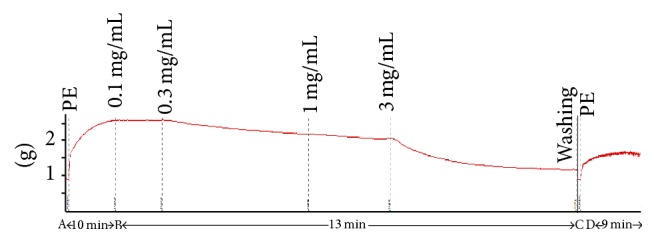
Representative tracing of dose-dependent relaxations (B-C) induced by cumulative additions of DCM (0.1–3 mg/mL) in PE-precontracted (A-B) aortic ring. Doses of DCM were only added once the relaxant response had stabilised. At the end of the dose-response experiment, the ring was allowed to equilibrate before adding PE (D) again to the tissue chamber to test for viability of the ring. *x*-axis: compressed passage of time.

All fractions partitioned with organic solvents (HX, DCM, EA, and BU) were able to significantly (*p* < 0.05) attenuate the PE-induced contractions of both endothelium-intact and -denuded aortic rings in a dose-dependent manner (Figure 1). Vehicle alone did not elicit any response. Although HX, DCM, EA, and BU all had vasodilatory effects and HX appeared to be more potent in intact preparation, only DCM was subjected to further fractionation as it was also found to be the most potent fraction in relaxing rat corpus cavernosum in our previous study [31]. At the end of each experiment, all tissues were found to be viable as contractions could still be induced by PE (Figure 2).

#### 3.2.2. Effects of DCM (DCM-I to DCM-VI) Subfractions on PE-Precontracted Aortic Ring

**Figure 3:**
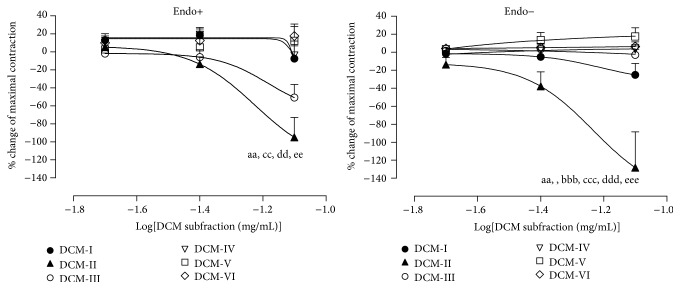
Effects of DCM subfractions on PE-precontracted endothelium-intact (Endo+) and endothelium-denuded (Endo−) aortic rings. Values are mean ± SEM (*n* = 6). ^aa^ *p* < 0.01 compared with DCM-I, ^bbb^ *p* < 0.001 compared with DCM-III, ^cc^ *p* < 0.01, ^ccc^ *p* < 0.001 compared with DCM-IV, ^dd^ *p* < 0.01, ^ddd^ *p* < 0.001 compared with DCM-V, ^ee^ *p* < 0.01, and ^eee^ *p* < 0.001 compared with DCM-VI.

Subfractions DCM-II and DCM-III were able to induce relaxations in PE-precontracted endothelium-intact aortic rings. However, in the PE-precontracted endothelium-denuded aortic rings, only DCM-II was able to induce relaxation. As shown in Figure 3, at a dose of 0.08 mg/mL, the relaxation caused by DCM-II in the intact preparation was significantly (*p* < 0.01) greater than DCM-I and DCM-IV to DCM-VI, while the relaxation caused by DCM-II in the denuded preparation was significantly greater than all the other DCM subfractions [DCM-I (*p* < 0.01) and DCM-III to DCM-VI (*p* < 0.001)]. Since DCM-II was the most potent subfraction that can bring about vasodilation, it was selected for all subsequent studies on mechanisms of action in aortic rings.

### 3.3. Mechanisms of Action of DCM-II on Aortic Ring

#### 3.3.1. Effects of DCM-II on Ang I- or Ang II-Induced Contractions

**Figure 4:**
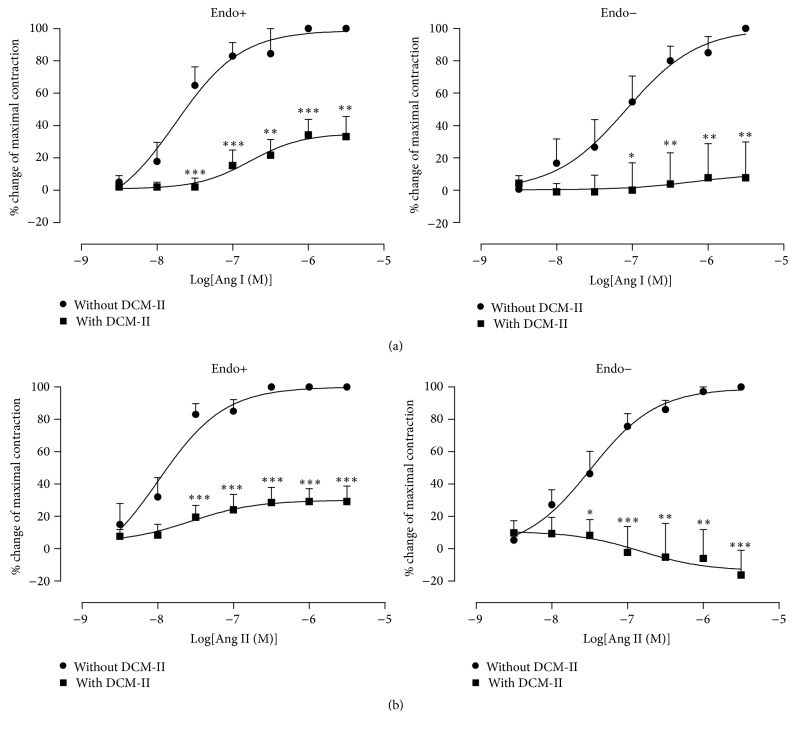
Effects of DCM-II pretreatment on (a) Ang I- or (b) Ang II-induced contractions in endothelium-intact (Endo+) and endothelium-denuded (Endo−) aortic rings. Values are mean ± SEM (*n* = 6). ^*∗*^ *p* < 0.05, ^*∗∗*^ *p* < 0.01, and ^*∗∗∗*^ *p* < 0.001 compared with control (without DCM-II).

Pretreatment of endothelium-intact and -denuded aortic rings with DCM-II significantly (*p* < 0.05) reduced the Ang I-induced contractions (Figure 4(a)). Furthermore, Ang II-induced contractions were also significantly (*p* < 0.05) reduced in the presence of DCM-II (Figure 4(b)). There was no significant difference in the responses elicited in endothelium-intact and -denuded rings that were pretreated with DCM-II.

#### 3.3.2. Effect of DCM-II on Ang II-Induced Contractions in PE-Precontracted Aortic Ring

**Figure 5:**
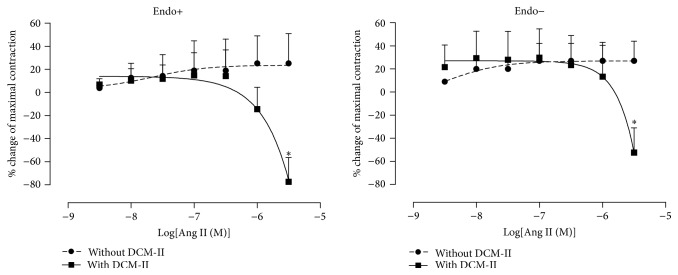
Effects of DCM-II pretreatment on Ang II-induced contractions in PE-precontracted endothelium-intact (Endo+) and endothelium-denuded (Endo−) aortic rings. Values are mean ± SEM (*n* = 6). ^*∗*^ *p* < 0.05 compared with control (without DCM-II).

In the absence of DCM-II, Ang II was able to elicit further contractions in both PE-precontracted intact and denuded aortic rings. However, in the presence of DCM-II, not only were the Ang II-induced contractions abolished, but significant (*p* < 0.05) further relaxation was observed in both endothelium-intact and -denuded rings (Figure 5).

#### 3.3.3. Effects of Antagonists of AT_2_R (PD 123319) and Mas Receptor (A-779) on Ang II-Induced Relaxation in PE-Precontracted Aortic Rings in the Presence of DCM-II

**Figure 6:**
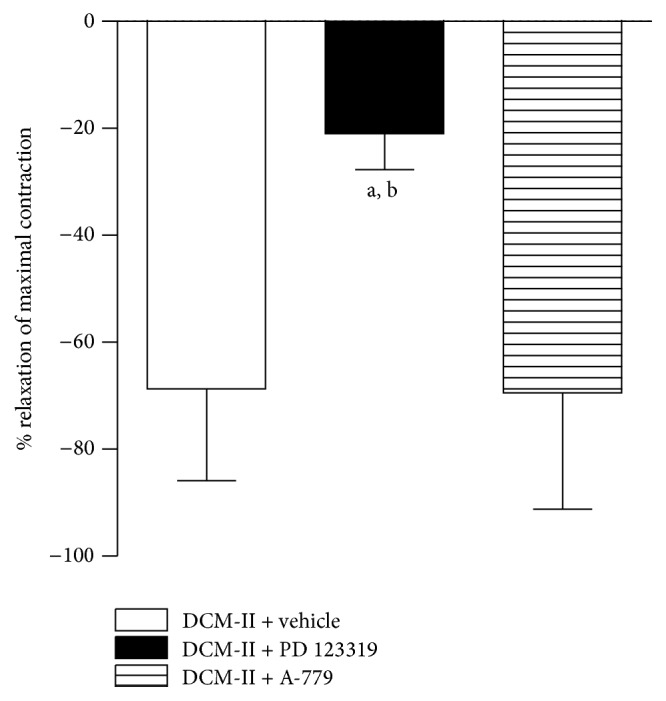
Effects of PD 123319 and A-779 on Ang II-induced relaxation in PE-precontracted endothelium-denuded aortic rings in the presence of DCM-II. Values are mean ± SEM (*n* = 6). ^a^ *p* < 0.05 compared with vehicle (control) and ^b^ *p* < 0.05 compared with A-779.

In the presence of the AT_2_R blocker, PD 123319, Ang II-induced relaxation was significantly (*p* < 0.05) attenuated. However, blockade of the Mas receptor by the antagonist A-779 did not affect the Ang II-induced relaxation (Figure 6).

#### 3.3.4. Effect of DCM-II on BK-Induced Relaxations in PE-Precontracted Aortic Ring

**Figure 7:**
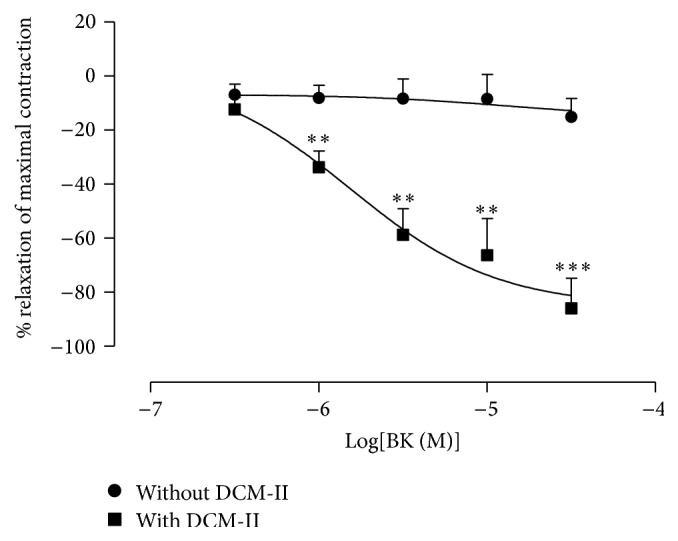
Effect of DCM-II pretreatment on BK-induced relaxations in PE-precontracted endothelium-intact aortic rings. Values are mean ± SEM (*n* = 6). ^*∗∗*^ *p* < 0.01 and ^*∗∗∗*^ *p* < 0.001 compared with control (without DCM-II).

As shown in Figure 7, BK-induced relaxations were significantly (*p* < 0.01) enhanced in endothelium-intact aortic rings that were pretreated with DCM-II.

### 3.4. *In Vitro* Studies

#### 3.4.1. Effect of DCM-II on ACE Activity

**Figure 8:**
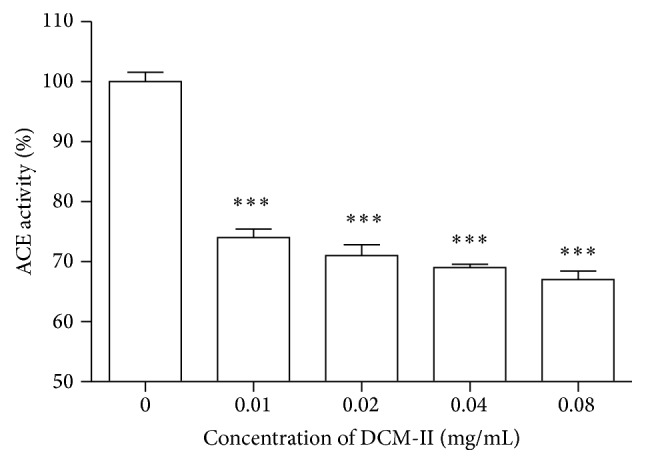
Effect of DCM-II on activity of ACE. Values are mean ± SEM (*n* = 3). ^*∗∗∗*^ *p* < 0.001 compared with control (vehicle only).

Figure 8 shows that the activity of ACE was significantly (*p* < 0.001) inhibited in the presence of DCM-II in a dose-dependent manner.

## 4. Discussion

The results presented here clearly demonstrate a novel finding that root extracts of* E. longifolia* contain putative compounds that could induce vasodilation (Figure 1). Moreover, the study shows that DCM-II, which is a more purified form of the root extracts, is able to bring about the vasodilation (Figure 3)* via* dual inhibition of AT_1_R and ACE as well as enhancement of BK effects. The putative compounds present in DCM-II appear to be semipolar in nature. The precise molecule involved awaits further investigation.

As shown in Figure 4(a), Ang I alone was able to cause dose-dependent vasocontractions in both endothelium-intact and -denuded aortic rings indicating the presence of tissue ACE and that removal of the endothelium does not prevent the local conversion of physiologically inert Ang I [32] to vasoconstrictive Ang II by the tissue ACE, similar to findings reported elsewhere [33, 34]. Pretreatment with DCM-II significantly decreased the Ang I-induced vasocontractions in both endothelium-intact and -denuded rings with no significant difference in the degree of inhibition between the intact and denuded rings (Figure 4(a)). These observations reflect that DCM-II might be able to inhibit vascular ACE, thus reducing the local formation of newly converted Ang II.

It is well established that Ang II is a powerful vasoconstrictor as demonstrated in Figure 4(b). Nonetheless, pretreatment of both endothelium-intact and -denuded aortic rings with DCM-II could significantly reduce the Ang II-induced contractions (Figure 4(b)), suggesting that DCM-II could antagonise the contractile effect of Ang II besides being able to inhibit the formation of Ang II (Figure 4(a)). Indeed, pretreatment of intact and denuded rings with DCM-II not only abolished the Ang II-induced contractions, but also could result in Ang II-induced relaxations (Figure 5). In addition, these Ang II-induced relaxations appeared to be independent of endothelium as there was no significant difference in the vasorelaxant effects observed in intact or denuded rings. These results concur with the endothelium-independent vasodilatory effects of Ang II observed in rat aortae [35] and mesenteric microvessels [36]. It is generally accepted that Ang II elicits diverse physiological effects through different types of Ang II receptor. Stimulation of AT_1_R would cause prolonged increase in BP due to vasoconstriction [17] while activation of AT_2_R would reduce BP because of vasodilation [37]. However, Ang II activation of AT_2_R is only manifested in the presence of AT_1_R blockade due to the higher affinity of Ang II to AT_1_R and the lower density of AT_2_R [29]. In particular, the study of Fukada et al. [35] demonstrated that Ang II-induced endothelium-independent relaxation of rat aorta is mediated* via* AT_2_R located in VSM. Thus, the relaxations observed when Ang II was added to PE-precontracted rings that had been pretreated with DCM-II (Figure 5) could be mostly due to stimulation of AT_2_R in VSM after AT_1_R had been saturated by DCM-II during the incubation. Indeed, in the presence of DCM-II, Ang II-induced relaxation was found to be significantly reduced when the AT_2_R was blocked by PD 123319 but remained unaffected when the Mas receptor was blocked by A-779 (Figure 6). These observations strongly suggest that the Ang II-induced relaxation could be brought about by direct activation of the AT_2_R rather than through the conversion of Ang II by angiotensin-converting enzyme 2 (ACE 2) to angiotensin (1–7) (Ang (1–7)) that binds to the Mas receptor. Hence, this finding of an endothelium-independent vasodilatory effect of DCM-II may prove to be of immense significance in the process of drug discovery, as the development of hypertension is highly associated with endothelial dysfunction [38].

As a counterregulatory system to the RAS, the kallikrein-kinin system produces BK that causes vasodilation by binding mainly to B_2_ receptors [18] and only in the absence of B_2_ receptors that BK may bind to the B_1_ receptors [39, 40]. In this investigation, DCM-II was found to be able to enhance the relaxations induced by BK in PE-precontracted endothelium-intact aortic rings (Figure 7). This could probably be due to the ACE-inhibitory property of DCM-II as ACEi are able to potentiate the actions of BK by lowering the rate of degradation and increasing the sensitivity of B_2_ receptors [41], as well as direct activation of B_1_ receptors to release nitric oxide [42–44]. Thus, an* in vitro* study was carried out to verify the ACE-inhibitory property of DCM-II, and indeed DCM-II was found able to significantly inhibit the activity of ACE in a dose-dependent manner (Figure 8). Moreover, inhibition of ACE leading to the conversion of Ang I by ACE 2 to produce angiotensin (1–9) (Ang (1–9)) could confer further beneficial effects on the cardiovascular system [45], as evidenced by a recent report demonstrating that Ang (1–9) could cause endothelial nitric oxide-dependent relaxation* via* activation of AT_2_R in rat aortic rings [46].

## 5. Conclusions

The present study conclusively demonstrates that the DCM-II subfraction obtained from the ethanolic root extract of* E. longifolia* is able to cause vasodilation. The vasodilatory effect of DCM-II is apparently due to antagonism of Ang II-induced contractions mediated* via *inhibition of ACE and AT_1_R. Moreover, ACE inhibition also potentiates the action of BK while AT_1_R blockade would unmask AT_2_R for stimulation to bring about vasodilation. These novel findings are important as they may present an alternative treatment for hypertension that would complement the conventional therapies available at present. The discovery that DCM-II is also effective in relaxing the corpus cavernosum (manuscript submitted and under review) could auger well for the development of a therapy aimed at hypertensive patients with ED or at those who are on conventional antihypertensive drugs suffering from ED as a side-effect. It is pivotal that the quality of life in these individuals is maintained to ensure compliance and adherence to their antihypertensive treatment. As far as can be ascertained, this is the first report of such findings for this plant.

## Additional Points

*Justification of the Importance of the Work*. This study is the first to report of a vasodilatory effect of a partially purified subfraction of root extracts of* Eurycoma longifolia* Jack with the mechanisms of action underlying the vasodilatory effect being elucidated. These novel findings are important as they may offer an alternative to complement the conventional therapies for the control of hypertension. This may be of particular benefit to individuals who experience side-effects with or who are intolerant of or not responsive to the conventional treatments. Furthermore, since the subfraction under investigation has also been found to effectively cause relaxation in the corpus cavernosum (manuscript submitted and under review), it is anticipated that this subfraction could be developed into an antihypertensive treatment that would be able to preserve or improve erectile function in hypertensive patients, especially in those where erectile dysfunction poses a major concern. It is pivotal that the quality of life is maintained so as to ensure compliance in continuing and adhering to their prescribed treatments.
